# The HKT1 Na^+^ transporter protects plant fertility by decreasing Na^+^ content in stamen filaments

**DOI:** 10.1126/sciadv.adg5495

**Published:** 2023-06-02

**Authors:** Takeshi Uchiyama, Shunya Saito, Taro Yamanashi, Megumi Kato, Kosuke Takebayashi, Shin Hamamoto, Masaru Tsujii, Tomoko Takagi, Noriko Nagata, Hayato Ikeda, Hidetoshi Kikunaga, Toshimi Suda, Sho Toyama, Misako Miwa, Shigeo Matsuyama, Mitsunori Seo, Tomoaki Horie, Takashi Kuromori, Mutsumi Yamagami, Yasuhiro Ishimaru, Nobuyuki Uozumi

**Affiliations:** ^1^ Department of Biomolecular Engineering, Graduate School of Engineering, Tohoku University, Sendai 980-8579, Japan.; ^2^ Department of Chemical and Biological Sciences, Faculty of Science, Japan Women’s University, Bunkyo-ku, Tokyo 112-8681, Japan.; ^3^ Research Center for Electron Photon Science, Tohoku University, Sendai 982-0826, Japan.; ^4^ Cyclotron and Radioisotope Center, Tohoku University, Sendai 980-8578, Japan.; ^5^ Quantum Science and Energy Engineering, Graduate School of Engineering, Tohoku University, Sendai 980-8579, Japan.; ^6^ RIKEN Center for Sustainable Resource Science, Yokohama 230-0045, Japan.; ^7^ Division of Applied Biology, Faculty of Textile Science and Technology, Shinshu University, Ueda 386-8567, Japan.; ^8^ Advanced Science Research Center, Okayama University, Okayama 700-8530, Japan.; ^9^ Institute for Environmental Sciences, Aomori 039-3212, Japan.

## Abstract

Salinity stress can greatly reduce seed production because plants are especially sensitive to salt during their reproductive stage. Here, we show that the sodium ion transporter AtHKT1;1 is specifically expressed around the phloem and xylem of the stamen in *Arabidopsis thaliana* to prevent a marked decrease in seed production caused by salt stress. The stamens of AtHKT1;1 mutant under salt stress overaccumulate Na^+^, limiting their elongation and resulting in male sterility. Specifically restricting *AtHKT1;1* expression to the phloem leads to a 1.5-fold increase in the seed yield upon sodium ion stress. Expanding phloem expression of AtHKT1;1 throughout the entire plant is a promising strategy for increasing plant productivity under salinity stress.

## Teaser

The introduction of Na^+^ transporter that functions in phloem of stamens can increase seed yield under saline conditions.

## INTRODUCTION

Sodium ion (Na^+^) is not an essential element for growth and reproduction of terrestrial plants except for C_4_ plants (*1*). Even more, excess salt is one cause of abiotic stress that is devastating to agricultural productivity. More than 4.4% of topsoil and more than 8.7% of subsoil of the total global land area across 118 countries are estimated to be salt-affected soils (*2*), and the salt-affected area is increasing annually by 10% (*3*, *4*). To meet the food needs of the world’s population, plant productivity studies in salt-affected environments are of great importance.

The level of sensitivity of plants to salinity stress depends on the plant’s developmental stage, such as germination, vegetative, and reproductive stage (*5*, *6*). In chickpea (*Cicer arietinum* L.), a salt-sensitive plant, the reproductive stage is more sensitive to salinity than the vegetative growth stage. More specifically, the salinity has detrimental effects on stigma viability, pollen germination, and pollen tube growth, which become more severe during the conversion from flowers to seed pods (*6*). Rice is also highly sensitive to salinity during its reproductive stage (*7*). Pollen and stigma viability is severely affected by treatment with high concentrations of NaCl in rice. During the sexual reproduction stage, in response to excess salt, small metabolites such as proline accumulate in large amounts to protect the pollen against osmotic stress and cytotoxicity caused by salinity (*8*). These protection mechanisms that are active during the highly salt-sensitive reproductive stage ensure the fertility of the plant. Several factors leading to salinity-delayed flowering have been identified in *Arabidopsis*, for instance, DELLA proteins (*9*). In contrast, WRKY71 speeds up flowering as a mechanism to enable the plant to complete its reproductive stage and avoid salt stress damage (*10*). Nevertheless, it remains to be identified whether other molecular mechanisms underlying salt tolerance are active during the reproductive growth stage of *Arabidopsis*. Deciphering these molecular mechanisms will be essential for understanding salt tolerance mechanisms and developing strategies to increase plant productivity (*11*).

Plants have developed mechanisms to keep Na^+^ concentrations inside the plant at harmless levels, those include controlling the Na^+^/K^+^ balance, Na^+^ extrusion, and Na^+^ sequestration (*12*, *13*). In *Arabidopsis thaliana* (henceforth *Arabidopsis*), Na^+^ export from the cytosol is performed by Na^+^/H^+^ antiporters, AtSOS1 (AtNHX7) located in the plasma membrane, and AtNHX1 in the vacuole membrane; their activity leads to a decrease in the cytosolic Na^+^ concentration (*14*, *15*). A different type of Na^+^ transport system, Na^+^ transport system, AtHKT1;1, which is involved in conferring Na^+^ tolerance, has been identified (*16*–*19*). High affinity K transporters (HKTs) belong to the Trk/Ktr/HKT family, a K^+^ and/or Na^+^ uptake system that is found in a wide range of organisms (*20*, *21*). During the vegetative stage, AtHKT1;1 function is important in xylem parenchyma cells and for Na^+^ unloading from xylem in roots (*18*, *19*). In the *athkt1* mutant, damage due to high Na^+^ accumulation is more pronounced in the shoot than in the roots (*17*–*19*, *22*, *23*). In the past two decades, this function in roots has been exploited for the generation of plants whose vegetative stage is tolerant to high salt stress. The function of AtHKT1;1 in flower development and seed production remains to be investigated although *AtHKT1;1* transcripts are observed in phloem in leaves (*19*, *22*, *23*).

## RESULTS

### The *athkt1* mutation confers male sterility under salinity conditions

**Fig. 1.**
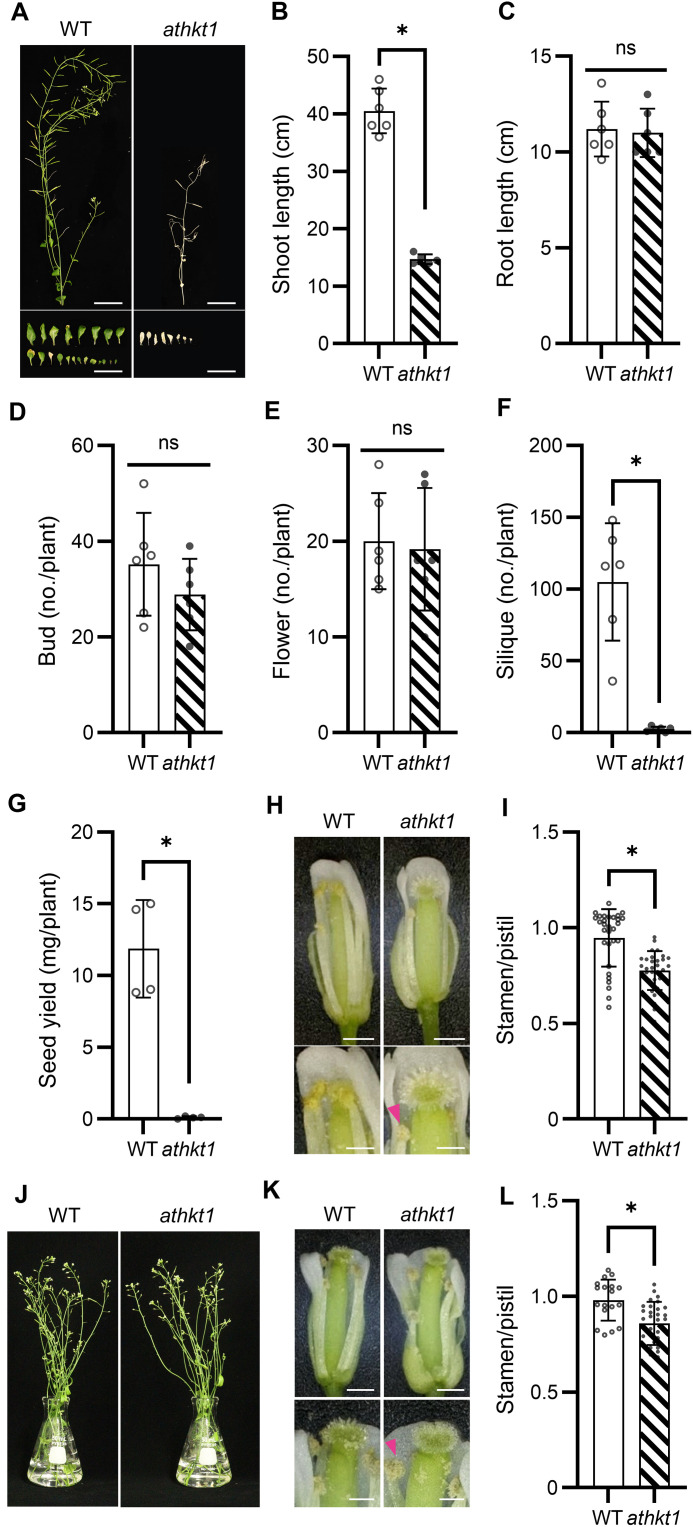
Phenotypes of flowers from the *athkt1* mutant. (**A**) Growth and reproduction were inhibited by salt treatment (30 mM NaCl) in the WT and the *athkt1* mutant. Shoot and rosette leaves of a typical plant are shown. Scale bars, 3 cm. (**B**) Shoot length, (**C**) root length, and numbers of (**D**) buds, (**E**) flowers, and (**F**) siliques. Data are from three independent experiments. Values are the means ± SD; *n* = 6. (**G**) Seed yield. Data are from three independent experiments. *n* = 4. (**H**) Phenotype of flowers of the WT and the *athkt1* mutant. The magenta arrowhead points to the short stamen of the *athkt1* mutant. Scale bars, 500 μm (top) and 250 μm (bottom). (**I**) Ratio of length of stamens to pistil of the plants shown in (H). *n* = 30. (**J**) Phenotypes of cut flower stalks. (**K**) Phenotypes of flowers from excised flower stalks shown in (J). The magenta arrowhead points to the short stamen of the *athkt1* mutant. Scale bars, 500 μm (top) and 250 μm (bottom). (**L**) Ration of length of stamens to pistil of flower stalks shown in (J). *n* = 18 to 30. Student’s *t* test, *P < 0.05. ns, not significant.

We tested the effect of salinity stress on the reproductive stages of the *athkt1* mutant. At 1 day before bolting (hereinafter referred to as the prebolting stage), 30 mM NaCl was added to the medium of the wild type (WT) and the *athkt1* mutant growing in hydroponic culture (Fig. 1A). This elevated salinity condition reduced the overall growth of the *athkt1* mutant compared with that of the WT, which had previously been observed for plants cultured in soil (*16*, *17*, *22*). The shoots of the *athkt1* mutant were shorter than those of the WT (Fig. 1B), whereas the length of the roots and the number of buds and flowers in the *athkt1* mutant were similar to those of the WT (Fig. 1, C to E). However, the number of siliques and the seed yield of the *athkt1* mutant were strongly reduced under salt conditions (Fig. 1, F and G). To further investigate morphological changes occurring during the development from flower to silique, flowers at the pollination stage [stages 14 and 15, according to (*24*)] were examined. The stamens (consisting of anther and filament) of the *athkt1* mutant were shorter than those of the WT (Fig. 1, H and I). To test the possibility that AtHKT1;1-dependent acquired salt tolerance is not solely accomplished by its function in roots (*18*), we conducted another experiment using cut flower stalks placed into medium with 3 mM NaCl (Fig. 1, J to L). The ratio of stamen/pistil length was reduced in the *athkt1* mutant treated with salt stress, but not in the WT. The same experiments were performed using the Wassilewskija (Ws) ecotype, used in other studies of AtHKT1;1 (*17*, *19*, *25*). The response of the *athkt1* mutant in the Ws background (fig. S2, A to I) was similar to that of the *athkt1* mutant in the Col-*gl* background (Fig. 1 and fig. S1). Another mutant with impaired filament elongation is *atgtr1*, a mutant of the AtGTR1 glucosinolate transporter, whose phenotype results in low gibberellin content in the flowers (*26*). We determined the gibberellin content in flowers of *athkt1* and found that it was not different from that of WT flowers (fig. S3). These results suggested that AtHKT1;1 was required for stamen elongation and seed production under salt stress conditions.

### AtHKT1;1 is simultaneously expressed in companion cells and xylem parenchyma cells in the filament

**Fig. 2.**
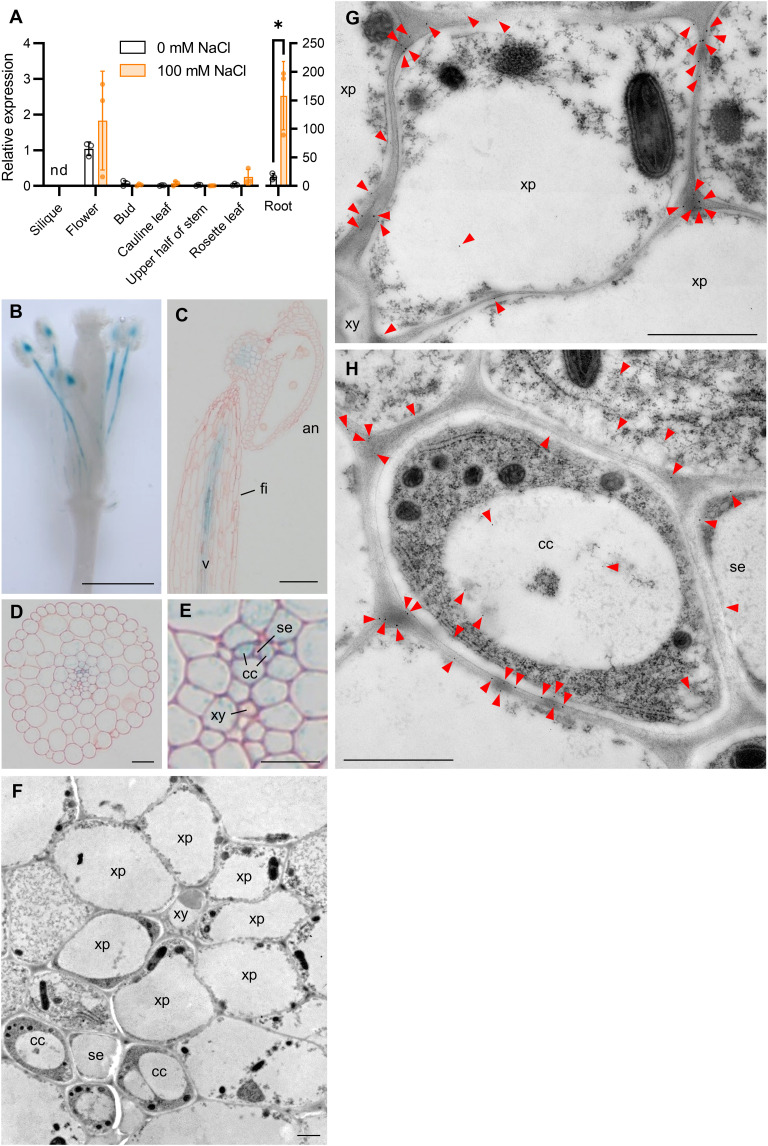
Localization of AtHKT1;1 in floral organs. (**A**) Quantification of *AtHKT1;1* transcripts in various organs. White bars, control; orange bars, 100 mM NaCl. *n* = 3; Student’s *t* test, *P < 0.05. nd, not determined. (**B** to **E**) GUS staining of the flowers without sepals and petals (B), stamen (C), cross section of filament (D). (E) Magnification of part of the filaments in (D). an, anther; fi, filament; se, sieve element; cc, companion cell; ph, phloem; xy, xylem; v, vascular bundle. Scale bars, 1 mm (B), 100 μm (C), 20 μm (D), and 10 μm (E). (**F** to **H**) Cross sections of *A. thaliana* filament were immunostained using an anti-AtHKT1;1 antibody (F). Gold particle–labeled AtHKT1;1 [arrowheads in (G) and (H)] was detected in xylem parenchyma cells (xp) (G) and companion cells (cc) (H). Scale bars, 1 μm.

We examined *AtHKT1;1* expression in different plant tissues using reverse transcription quantitative polymerase chain reaction (RT-qPCR) (Fig. 2A). *AtHKT1;1* expression was confirmed in roots as described previously (*27*), but abundant transcript was also observed in flowers, at a much higher level than in other tissues. To assess tissue specificity of *AtHKT1;1* expression, histochemical assays were conducted with plants expressing the β*-*glucuronidase (GUS) gene under control of 5.4 kb of the *AtHKT1;1* promoter (*23*, *28*, *29*). GUS staining was seen in vascular bundles of the anther bases and filaments (Fig. 2, B to D). In particular, strong staining was seen in the companion cells of the phloem of the filaments (Fig. 2E). No difference in staining was observed between salt-treated plants and untreated plants (fig. S4). To confirm these findings, we localized AtHKT1;1 in the filament of salt-treated plants by immunoelectron microscopy using an anti-AtHKT1;1 antibody (Fig. 2, F to H) (*19*). The immunological signals were detected in the plasma membrane of companion cells and that of xylem parenchyma cells (Fig. 2, G and H). These results suggested that AtHKT1;1 in the filament takes up Na^+^ from the xylem into xylem parenchyma and into the phloem via the companion cells.

### The *athkt1* mutant accumulates excess Na^+^ in the filament under salinity stress conditions

**Fig. 3.**
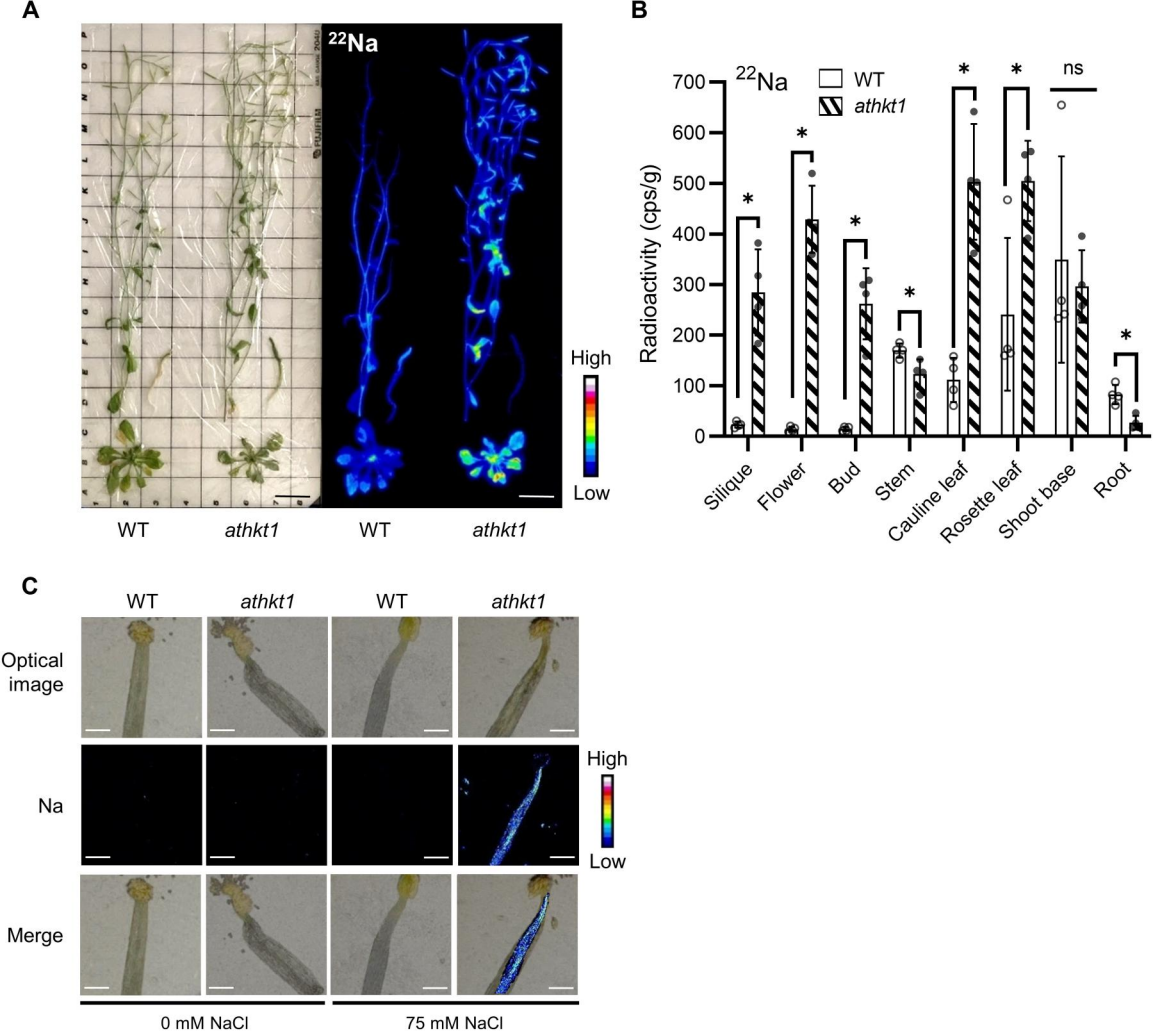
Distribution of Na^+^ during the reproductive stage of the *athkt1* mutant. (**A**) Representative autoradiographs of the WT and the *athkt1* mutant incubated with ^22^Na in the presence of 75 mM NaCl. Scale bars, 3 cm. (**B**) Quantification of the radioisotopes in various organs in ^22^Na-treated plants. Data are from three independent experiments. *n* = 4; Student’s *t* test, *P < 0.05. cps, count per second. (**C**) Elemental maps of Na in stamens of the WT and the *athkt1* mutant cultured as in (A) obtained by micro-PIXE analysis. Scale bars, 200 μm. Data are from three independent experiments.

To evaluate Na^+^ and K^+^ distribution in *athkt1* plants during the reproductive stage, we performed radioisotope tracer studies with ^22^Na and ^43^K. WT and *athkt1* plants grown until 2 weeks after bolting in hydroponic medium (Fig. 3A) were incubated for 2 days with 500 kilobecquerel (kBq) of ^22^Na plus 75 mM NaCl. Compared to the WT, the *athkt1* plants accumulated more ^22^Na in the apical tissues of siliques, flowers, buds, cauline leaves, and rosette leaves and less ^22^Na in stems and roots (Fig. 3, A and B). To rule out the possibility that loss of *AtHKT1;1* affected K^+^ translocation in plants, we performed absorption experiments with ^43^K. Consistent with Na^+^-specific transport of AtHKT1;1, no significant difference in ^43^K accumulation was observed in the WT and the *athkt1* mutant (fig. S5, A and B). On the basis of these results (Fig. 3, A and B), we investigated the distribution of Na^+^ and K^+^ in the stamens of the WT and the *athkt1* mutant in more detail using micro–particle-induced x-ray emission (micro-PIXE) analysis (*30*). When the plants were labeled in the same manner as described, Na^+^ signals were observed in the entire filament in the *athtk1* plants, but not in the WT (Fig. 3C). In contrast, K^+^ was distributed similarly in the WT and the *athkt1* mutant, regardless of whether they were treated with 75 mM NaCl (fig. S5C). These data showed that under high Na^+^ conditions, AtHKT1;1 contributed to the removal of excess Na^+^ from the filament.

### Na^+^-specific HKTs rescue the salt sensitivity of the *athkt1* mutant

**Fig. 4.**
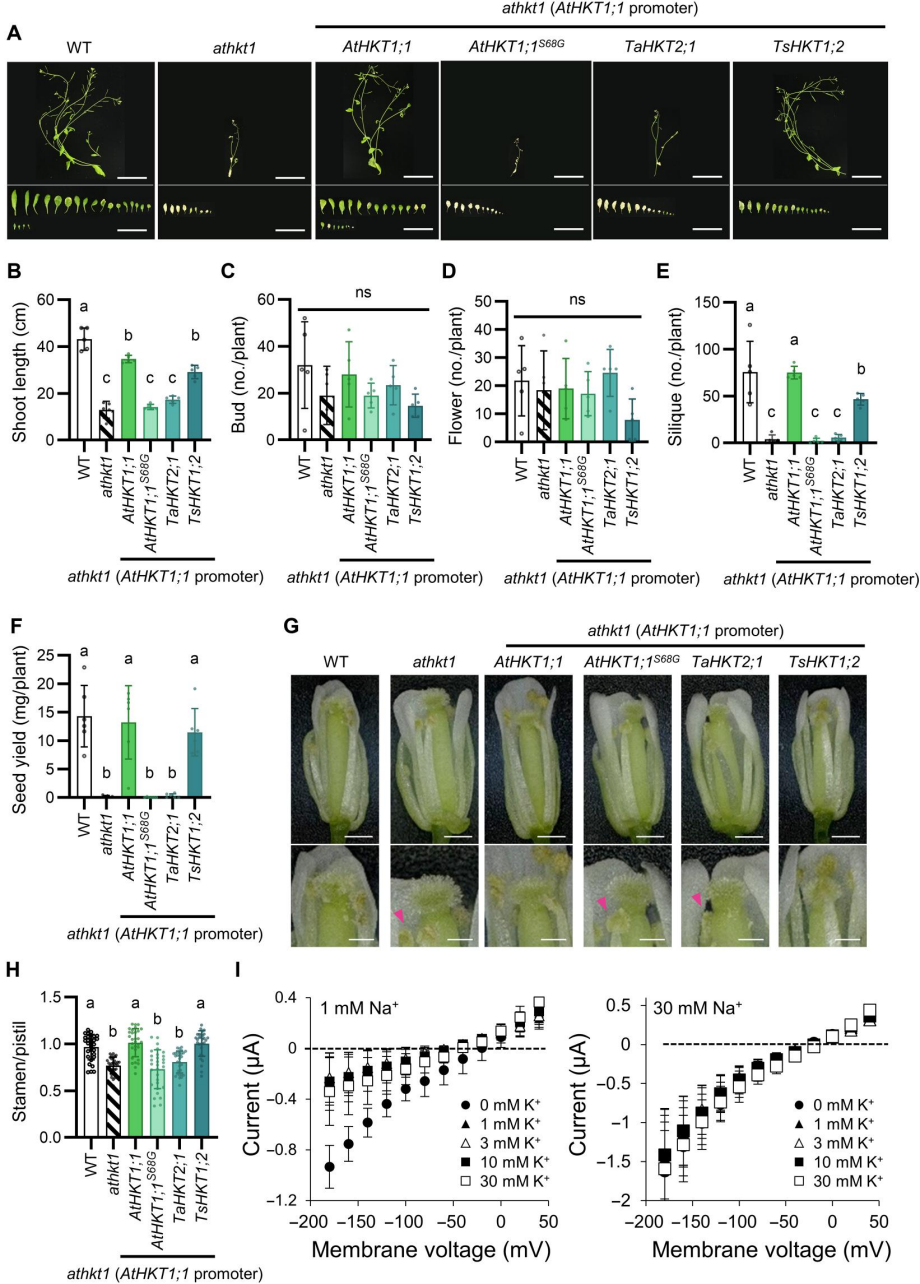
Phenotype of the *athkt1* mutant transformed with different HKTs. (**A**) Phenotype of the WT, *athkt1* mutant, and *P_AtHKT1;1_*-*HKT*s/*athkt1*. Shown are shoot and rosette leaves of a typical plant. Scale bars, 5 cm. (**B** to **E**) Shoot length (B) and numbers of buds (C), flowers (D), and siliques (E) are shown. Data are from three independent experiments. Values are the means ± SD; *n* = 4 to 5; one-way analysis of variance (ANOVA) with Tukey-Kramer test, *P* < 0.05. (**F**) Seed yield. Data are from three independent experiments. *n* = 4 to 6. (**G**) Phenotypes of the flowers. The magenta arrowheads point out the short stamens. Scale bars, 500 μm (top) and 250 μm (bottom). (**H**) Ratio of length of stamens to pistil of plants shown in panel (G). *n* = 30. (**I**) Changes in K^+^ dependency of TsHKT1;2 current at different Na^+^ concentrations. In the presence of either 1 mM NaCl or 30 mM NaCl, instantaneous current values (values at 50 ms after initiation of the test pulse) measured at the corresponding membrane voltage were plotted for different KCl concentrations. *n* = 3.

We tested whether reintroduction of HKT1 homologs can restore the salt sensitive phenotype of the *athkt1* mutant. AtHKT1;1 and TsHKT1;2 (from *Thellungiella salsuginea*) as representatives of subfamily 1, TaHKT2;1 as a representative of subfamily 2 and the point mutant AtHKT1;1^S68G^, which carries a mutation of a glycine in the selectivity filter, were expressed in the *athkt1* mutant under control of the *AtHKT1;1* promoter (5.4 kb) (*31*). We compared the phenotype of the *athkt1* mutant expressing AtHKT1;1, AtHKT1;1^S68G^, TaHKT2;1, and TsHKT1;2 with the WT and the *athkt1* mutant. Plants at the prebolting stage were cultured in medium with or without 30 mM NaCl in hydroponic culture and analyzed as for Fig. 1, A to G (Fig. 4 and fig. S6). *P_AtHKT1;1_*-*AtHKT1;1*/*athkt1* and *P_AtHKT1;1_*-*TsHKT1;2*/*athkt1* complemented the salt hypersensitivity, specifically shoot length, number of siliques, and seed yield of the *athkt1* mutant (Fig. 4, A, B, E, and F). On the other hand, *P_AtHKT1;1_*-*AtHKT1;1^S68G^*/*athkt1* and *P_AtHKT1;1_*-*TaHKT2;1*/*athkt1* did not rescue these phenotypes. Under control conditions, without added Na^+^, there was no significant difference between the plant lines (fig. S6, A to F). The changes in stamen morphology occurred before pollination (Fig. 4G). We measured the ratio of stamen to pistil length (Fig. 4H). The stamens of *P_AtHKT1;1_*-*AtHKT1;1^S68G^*/*athkt1* and *P_AtHKT1;1_*-*TaHKT2;1*/*athkt1* were as short as those of the *athkt1* mutant, whereas the length of the stamens of *P_AtHKT1;1_*-*AtHKT1;1*/*athkt1* and *P_AtHKT1;1_*-*TsHKT1;2*/*athkt1* was similar to that of the WT stamens. None of these differences were seen in any of the lines when they were cultured without added Na^+^ (fig. S6, G and H). To understand whether the selectivity of HKTs for Na^+^ or K^+^ affects plant reproduction, we examined the detailed ion transport characteristics of the same HKT1 homologs as tested above. Two-electrode voltage clamp oocyte recordings confirmed that AtHKT1;1^S68G^ and TaHKT2;1 were capable of mediating both Na^+^ and K^+^ transport, whereas AtHKT1;1 displayed Na^+^-specific ion currents (*32*, *33*). In the presence of 1 mM or 30 mM Na^+^, addition of external K^+^ caused a slight positive shift in the reversal potential of TsHKT1;2-mediated instantaneous currents (approximately −50 mV at 10 mM KCl and approximately −40 mV at 30 mM KCl), which is indicative of K^+^ permeation (Fig. 4I). However, the degree of K^+^-mediated shift in the reversal potential was much smaller to that of Na^+^-mediated shift previously reported, supporting that TsHKT1;2 was more permeable to Na^+^ than K^+^, which is similar to AtHKT1;1 (*33*, *34*). Na^+^ import by TsHKT1;2 seemed to be interrupted when excess K^+^ was present at 1 mM external Na^+^ but not at 30 mM, which suggested that the Na^+^ transport ability of TsHKT1;2 was less affected by high Na^+^ treatment. Together, these data suggested that reintroduction of Na^+^ transport into the *athkt1* mutant by transformation with HKT1 homologs such as AtHKT1;1 and TsHKT1;2 that have less K^+^ transport activity alleviated the salt hypersensitivity and recovered seed production to WT levels.

### Phloem-specific expression of AtHKT1;1 and TsHKT1;2 rescued salt sensitivity of the *athkt1* mutant

AtHKT1;1 was also expressed in the companion cells of the filaments, which enhanced filament elongation under salt conditions (Figs. 1, 2, and 4). To assess the importance of expression of HKTs in the phloem for protection against high salinity stress, we placed AtHKT1;1, AtHKT1;1^S68G^, TaHKT2;1, and TsHKT1;2 under control of the *SUC2* promoter and expressed them in companion cells throughout the *athkt1* plants. The *SUC2* gene encodes a plasma membrane sucrose-H^+^ symporter that is expressed specifically in companion cells of *Arabidopsis* (*35*, *36*). We compared the phenotype of the *athkt1* mutant expressing AtHKT1;1, AtHKT1;1^S68G^, TaHKT2;1, and TsHKT1;2 under the same conditions as used for Fig. 4 (A to F) (fig. S7). *P_SUC2_*-*AtHKT1;1*/*athkt1* and *P_SUC2_*-*TsHKT1;2*/*athkt1* complemented the salt hypersensitivity of *athkt1*, specifically shoot length, number of siliques, and seed yield (fig. S7, B, E, and F). On the other hand, the number of flowers in *P_SUC2_*-*AtHKT1;1^S68G^*/*athkt1* and *P_SUC2_*-*TaHKT2;1*/*athkt1* exceeded that of the WT (fig. S7D), but silique development was strongly suppressed (fig. S7E). None of these differences were seen in any of the lines when they were cultured without added NaCl. The stamens in *P_SUC2_*-*AtHKT1;1^S68G^*/*athkt1* and *P_SUC2_*-*TaHKT2;1*/*athkt1* remained as short as those of the *athkt1* plants, whereas the length of the stamens in *P_SUC2_*-*AtHKT1;1*/*athkt1* and *P_SUC2_*-*TsHKT1;2*/*athkt1* was similar to that of the WT (fig. S7G). These findings were consistent with the observed ratio of stamen to pistil length (fig. S7H). These data indicated that AtHKT1;1- and TsHKT1;2-mediated Na^+^ transport in all companion cells mitigated salinity stress at the reproductive stage and consequently rescued seed yield (fig. S7F).

### Phloem-specific expression of AtHKT1;1 and TsHKT1;2 increased salt tolerance of seed production of *Arabidopsis* plants

**Fig. 5.**
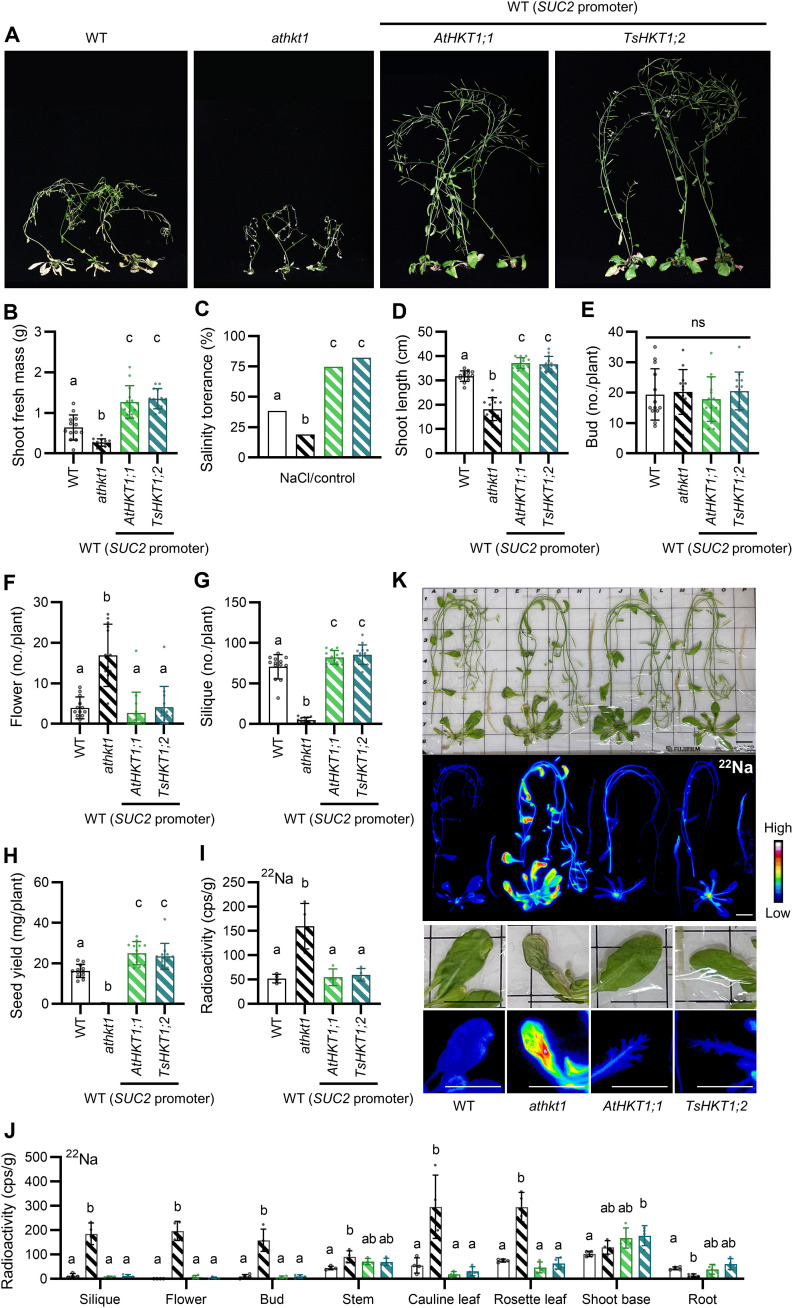
Acquisition of salt tolerance in plants expressing AtHKT1;1 or TsHKT1;2 in companion cells. (**A)** Phenotype of the WT, the *athkt1* mutant, and *P_SUC2_*-*HKT*s/WT. Shown are typical plants. (**B**) Fresh weight of shoots of plants grown under saline conditions. Data are from three independent experiments. *n* = 10 to 13. (**C**) Salt tolerance for the plant lines was calculated by expressing the fresh weight of shoots of plants under saline conditions as a percentage of the fresh weight of shoots of plants under control conditions, based on the data in (B). (**D** to **G**) Shoot length (D) and numbers of buds (E), flowers (F), and siliques (G) are shown. *n* = 10 to 13. (**H**) Seed yield. *n* = 9 to 12. (**I**) Radioactivity measured for whole plants of the WT, the *athkt1* mutant and *P_SUC2_*-*HKT*s/WT incubated with ^22^Na. (**J**) Radioactivity of various organs in ^22^Na-treated plants shown in (K). *n* = 4. (**K**) Representative autoradiographs of the WT, the *athkt1* mutant, and *P_SUC2_*-*HKT*s/WT treated with ^22^Na. Scale bars, 3 cm.

The results shown in fig. S7 suggested that AtHKT1;1 in the phloem can prevent stress due to excess Na^+^ during the reproductive stage. To corroborate this possible role and function of AtHKT1;1 in plants, we introduced *P_SUC2_*-*AtHKT1;1* and *P_SUC2_*-*TsHKT1;2* into the WT. Transformants were hydroponically grown until to the prebolting stage, and then the NaCl concentration was stepwise increased (30, 50, 75, 100 mM) every 5 days. Both lines exhibited increased growth under saline conditions (2.1-fold with *P_SUC2_*-*AtHKT1;1*/WT and 2.2-fold with *P_SUC2_*-*TsHKT1;2*/WT), compared with the WT (Fig. 5, A and B), while growth of the *athkt1* mutant was inhibited. *P_SUC2_*-*AtHKT1;1*/WT and *P_SUC2_*-*TsHKT1;2*/WT plants were more salt tolerant than the WT, determined on the basis of the ratio of shoot fresh weight of plants grown under high salt conditions relative to that of plants grown under control conditions (1.9-fold and 2.1-fold increase, respectively) (Fig. 5C). Specifically, the two lines showed increased shoot length, number of siliques, and seed yield (Fig. 5, D, G, and H). The numbers of buds and flowers and stamen development were the same as those of the WT (Fig. 5, E and F, and fig. S8). Seed yield of *P_SUC2_*-*AtHKT1;1*/WT and *P_SUC2_*-*TsHKT1;2*/WT increased 1.5-fold in both cases (Fig. 5H). None of these differences were seen in any of the lines when they were cultured without added Na^+^ (fig. S9). To further characterize the salt tolerance of *P_SUC2_*-*AtHKT1;1*/WT and *P_SUC2_*-*TsHKT1;2*/WT plants, we assessed the content and distribution of Na^+^ and K^+^ in the two lines using radioisotopes. ^22^Na plus 75 mM NaCl or ^43^K was applied to the two transformed lines, the WT and *athkt1* that had been grown for 2 weeks after bolting in hydroponic medium, the same as for Fig. 3 (Fig. 5, I to K, and fig, S10). ^22^Na content in whole plants was similar in the WT, *P_SUC2_*-*AtHKT1;1*/WT, and *P_SUC2_*-*TsHKT1;2*/WT, in contrast to the *athkt1* plants that showed higher levels overall (Fig. 5I). Tissue-specific ^22^Na accumulation showed subtle differences between the WT and the two transformed lines except for the base of the shoot, which accumulated more ^22^Na in *P_SUC2_*-*TsHKT1;2*/WT (Fig. 5J). ^43^K accumulation was the same for all (fig. S10A). The autoradiography images show uniform distribution of ^22^Na and ^43^K throughout the rosette leaves in the WT (Fig. 5K and fig. S10B). In contrast, ^22^Na signals were concentrated in the vascular bundles of the two transformed lines (Fig. 5K), but no such distinct focus was observed for the ^43^K signal (fig. S10B). The transformed lines expressing either AtHKT1;1 or TsHKT1;2 in the phloem appeared to have acquired more salt tolerance by collecting Na^+^ in the vascular bundles.

## DISCUSSION

Whether plants are sensitive or tolerant to salinity stress also depends on the lifecycle stages of growth/differentiation and tissues/organs (*1*). Silique formation with a low rate of seed abortion requires an adequate environment at the flowering stage (*37*). Considering that constitutive overexpression of *AtHKT1;1* driven by 35*S* promoter in the WT background resulted in a deleterious phenotype due to high shoot Na^+^ (*18*, *38*), it is clear that regulation of the tissue- and cell-specific and time-dependent expression of AtHKT1;1 is critical for Na^+^ detoxification. The results in this study shed light on the importance of the function of AtHKT1;1 in the phloem during the reproductive stage. AtHKT1;1 was expressed in the anther base and the vascular bundles of the filament, which contains sieve elements and companion cells as well as xylem parenchyma cells (Fig. 2). This accounts for the reduction in the filament elongation during salt stress observed in the *athkt1* mutant (Fig. 1). Introduction of *SUC2* promoter–driven *AtHKT1;1* restored the phenotype of the *athkt1* mutant back to the phenotype of the WT or the *athtk1* mutant expressing AtHKT1;1 under its own promoter (Fig. 4 and fig. S7). The AtHKT1;1 activity in companion cells throughout the plant facilitated Na^+^ retrieval into the phloem to mitigate salinity stress (Fig. 3C and fig. S7).

During the reproductive stage, removal of Na^+^ by AtHKT1;1 in the filament was required for filament elongation under salt conditions. These results are attributed to the expression of AtHKT1 in phloem companion cells in addition to xylem parenchyma cells (Fig. 2) (*19*, *22*, *23*). During the vegetative stage, AtHKT1;1 function is important in xylem parenchyma cells and for Na^+^ unloading from xylem in roots (*18*, *19*). In the *athkt1* mutant, damage due to high Na^+^ accumulation is more pronounced in the shoot than in the roots (*17*, *19*, *22*, *23*). Overexpression of AtHKT1;1 in the root stele, mediated by enhancer trap expression, decreases shoot Na^+^ accumulation and improves salinity tolerance (*18*, *38*). It appears that the contribution of salt tolerance by AtHKT1;1 varies depending on the expression site, in accordance with the developmental stage of the plant tissue.

We observed that Na^+^ was spread throughout the entire filament in the *athkt1* mutant, while no Na^+^ signal was observed in the WT (Fig. 3C). The xylem parenchyma cells and the companion cells where AtHKT1;1 was present were located next to each other (Fig. 2). This indicates that Na^+^ that is absorbed by the xylem parenchyma cells could be readily transferred to the companion cells in the filament. This close localization to AtHKT1;1 might help to minimize exposure of the cells in the stamen to Na^+^. This hypothesis explains well the finding that WT *Arabidopsis* plants acquired Na^+^ resistance when *SUC2* promoter–driven *AtHKT1;1* or *TsHKT1;2* was introduced into the WT (Fig. 5 and fig. S7). Phloem-specific expression of AtHKT1;1 or TsHKT1;2 in the WT kept ^22^Na^+^ in the vicinity of the vascular bundles when plants were grown in medium containing 75 mM NaCl, while ^22^Na^+^ diffused throughout the entire leaf in the WT (Fig. 5K). The results therefore showed an efficient translocation of Na^+^ from the xylem to the phloem, while preserving retrieval competence of Na^+^ from the xylem by AtHKT1;1. This notion also accounted for the complementation of the salt-sensitive phenotype of the *athkt1;1* mutant by introduction of *SUC2* promoter–driven AtHKT1;1 (Fig. 4). In the mutants, Na^+^ originating from the xylem can reach companion cells via two pathways: the apoplast or the symplast. In the apoplastic pathway, Na^+^ travels through extracellular spaces to reach the companion cells, whereas in the symplastic pathway, Na^+^ may be taken up via nonselective transporters and reach the companion cells through the plasmodesmata.

TaHKT2;1 and AtHKT1;1^S68G^, which also have K^+^ permeability and are less selective for Na^+^ over K^+^, failed to rescue the short stamen phenotype of the *athkt1* mutant under salinity (Fig. 4 and fig. S7). TaHKT2;1 belongs to subfamily 2, whose members have a glycine in the first pore region that is necessary for K^+^ transport activity (*34*). AtHKT1;1^S68G^ that has a glycine introduced at the corresponding site also has K^+^ transport activity (*34*). Although both transporters have Na^+^ transport activity, they clearly function as K^+^ uptake systems in yeast (*34*, *39*). TaHKT2;1-mediated Na^+^ uptake is controlled by K^+^ (*32*). Under physiological conditions in the presence of sufficient K^+^, Na^+^ transport activity seems to be relatively low, which may be the reason why both transporters were unable to complement the *athkt1* phenotype. On the other hand, TsHKT1;2 was more selectively permeable to Na^+^ than to K^+^ (Fig. 4I), which makes it more similar to AtHKT1;1 (subfamily 1) with regard to its Na^+^/K^+^ selectivity since both TsHKT1;2 and AtHKT1;1 have strong Na^+^ permeability with only low affinity for K^+^ (*27*, *38*). On the basis of our findings (Figs. 1 to 3 and 5 and fig. S7), another major physiological role of AtHKT1;1 in the companion cells is removal of Na^+^ from the stamen rather than maintaining Na^+^/K^+^ balance.

Enhancing salt tolerance in plants is a challenging task since abiotic stresses such as salt stress affect many aspects of plant physiology. There is evidence for a relationship between AtHKT1;1 activity and sugar metabolism during salt stress (*40*, *41*). Moreover, it is still unknown whether the short stamen filaments phenotype in the *athkt1* mutant was due to reduced cell elongation and/or cell division/numbers. Salt stress treatment of roots inhibits both cell division and elongation (*42*, *43*). The same may be true for the growth inhibition of stamens in this study (Fig. 1). TrkG, an ancestor of Trk/Ktr/HKT in *Escherichia coli*, can transport both Na^+^ and K^+^, and K^+^ transport assists cell growth (*21*). AtHKT1;1 also has K^+^ uptake transport activity (*27*), which supplies the major element K^+^ to the cells. Hence, the *athkt1;1* mutant may be more sensitive to Na^+^ inhibition in the filament. Nevertheless, the transport of Na^+^, which is the cause of salt damage, can be considered the most direct target for salt stress adaptation (*12*, *13*). Salt-tolerant plants have been generated by constitutive overexpression of SOS1 and NHX throughout the plant (*14*, *15*), as well as overexpression of AtHKT1;1 in the central column of the root (*18*). Furthermore, reports have indicated the presence of Ca^2+^ influx transporters that interact with Na^+^ upstream of the SOS1 pathway (*44*), implying the involvement of multiple transport systems in the response to salt stress through intracellular signaling pathways, including Ca^2+^ signaling. Coordination of expression of Na^+^ transporters in different plant parts may lead to the development of more effective salt-tolerant plants in the future.

## MATERIALS AND METHODS

### Plasmids and *Arabidopsis* transformation

A construct placing the GUS gene under control of the AtHKT1;1 promoter was created in three steps. First, a 5.412–base pair (bp) *AtHKT1;1* promoter fragment was obtained by PCR using BAC clone F24G24 (accession: AL049488) as template with a set of primers, 5′-CGACTAAGCTTCATTCTTGTGCAGC-3′ (Hind III site underlined) and 5′-ATGAGGACGTGAAGTGGTTCTTGG-3′. In a second step, the binary vector containing the GUS gene and 0.8 kb of the *AtHKT1;1* promoter (*17*) was digested with Hind III and Xho I, taking advantage of an Xho I site in the native promoter sequence, effectively removing the 0.8-kb promoter. In a third step, the 5.4-kb *AtHKT1;1* promoter from the first step was subcloned into the Hind III–Xho I sites of the binary vector containing the hygromycin resistance gene for plant selection. The final construct contained the GUS gene and the complete 5.4 kb of *AtHKT1;1* genome sequence immediately upstream from the start of *AtHKT1;1.*

To create constructs for the expression of different HKTs under control of the 5.4-kb *AtHKT1;1* promoter, we first had sequences synthesized that included 837 bp of the AtHKT1;1 promoter region seamless conjugated to cDNAs encoding AtHKT1;1, AtHKT1;1-S68G, TaHKT2;1, or TsHKT1;2 (TAKARA, Kusatsu, Japan). These sequences were cloned into the Hind III and Sac I sites in the pBI101 binary vector. In subsequent steps, Xho I–digested fragments (containing the HKT1 homolog) isolated from the resultant plasmids (2 to 3 kbp) were combined with the 5.4-kb Hind III–Xho I *AtHKT1;1* promoter region described above into the Hind III and Xho I sites of the pBlueScript II vector. Last, a Pst I– and Kpn I–digested fragment, containing the 5.4-kb *AtHKT1;1* promoter upstream of the HKT1 homolog, was isolated from pBlueScript II and cloned into pCAMBIA1300 binary vector.

To express HKTs under control of the 1.2-kb *SUC2* promoter, cDNAs encoding AtHKT1;1, AtHKT1;1-S68G (*34*), TaHKT2;1, and TsHKT1;2 (*38*) were amplified by PCR with the primer sets Asc I–AtHKT1–F (5′-ATAAGGGTGGGCGCGCCATGGACAGAGTGGTGGCA-3′); Sac I–AtHKT1–R (5′-GATCGGGGAAATTCGAGCTCTTAGGAAGACGAGGGGTA-3′); Asc I–AtHKT1–F, Sac I–AtHKT1–R, and Asc I–TaHKT1–F (5′-ATAAGGGTGGGCGCGCCATGGGCCGGGTGAAAAGA-3′); Sac I–TaHKT1–R (5′-GATCGGGGAAATTCGAGCTCTCATACTTTCCAGGATTT-3′); Asc I–TsHKT1–F (5′-ATAAGGGTGGGCGCGCCATGGAGAGAGTTGTGGAC-3′); and Sac I–TsHKT1–R (5′-GATCGGGGAAATTCGAGCTCTTACGAAGATGAAGGATA-3′), respectively. Each PCR product was cloned into the Asc I and Sac I sites of the *SUC2* promoter–nGFP vector (*36*). The plasmids were introduced into *Agrobacterium tumefaciens* GV3101 and then used for transformation of the *athkt1* mutant or the WT by floral dip (*45*).

### Plant growth

For the experiments described below, plants were grown hydroponically as follows. Sterilized seeds of *A. thaliana* were sown on rock wool in 1.5-ml centrifuge tubes with the bottoms cut off, suspended in hydroponic medium consisting of 2 mM KNO_3_, 1 mM NH_4_H_2_PO_4_, 1 mM MgSO_4_, 1 mM Ca(NO_3_)_2_·4H_2_O, 0.05 mM Fe[III]-EDTA, 0.3 μM CuSO_4_·4H_2_O, 1 μM ZnSO_4_·7H_2_O, 20 μM MnCl_2_·4H_2_O, 70 μM H_3_BO_3_, 0.2 μM K_2_MoO_4_, and 0.1 μM CoCl_2_, and vernalized for 3 days at 4°C. The plants were grown under a 16/8-hour light/dark cycle at 22°C with illumination (80 μmol/m^2^ per second). The medium was exchanged twice a week.

For salt sensitivity assays, *Arabidopsis* plants (WTs Col-*gl* and Ws, *athkt1*, *P_AtHKT1;1_*-*HKTs*/*athkt1*, or *P_SUC2_*-*HKTs*/*athkt1* T3 plants) at 1 day before bolting (referred to as the prebolting stage) were transferred to hydroponic medium with or without 30 mM NaCl and grown for an additional 2 or 3 weeks. Flower stage was determined using the criteria described by Smyth *et al.* (*24*). Length of stamens and pistils was measured from images of stage 15 flowers collected from at least four plants after 2 weeks of culture with or without NaCl, with the help of ImageJ software (http://rsbweb.nih.gov/ij). Shoot length, root length, buds (stages 6 to 12), flowers (stages 13 to 15), and siliques (stages 16 to 20) were measured after 3 weeks of culture with or without NaCl and cut flower stalks were dried for 3 to 4 weeks for measurement of seed yield. To specifically observe the phenotypes of flower stalks, stalks from plants grown in hydroponic medium for 2 weeks after bolting were cut off under water, their length was adjusted to 12 cm, and flowers and siliques were removed before placing them into medium with or without 3 mM NaCl for 5 days.

For salt tolerance analysis, *P_SUC2_*-*AtHKT1;1*/WT T3 plants, *P_SUC2_*-*TsHKT1;2*/WT T3 plants, the WT Col-*gl* and *athkt1* were cultured in the same manner except for the duration of the NaCl-treatment. Plants at the prebolting stage were grown on hydroponic medium with added NaCl at stepwise increasing concentrations (30, 50, 75, and 100 mM). Plants were grown for 5 days at each concentration, for a total of 15 or 20 days. Controls were grown on medium without added NaCl. Stage 15 flowers harvested after 15 days of culture were used to measure the length of all stamens and pistils. Plants grown for 20 days were used to measure shoot length, buds (stages 6 to 12), flowers (stages 13 to 15), siliques (stage 16 to 20), and fresh weight of excised shoots. The shoots were then dried for 3 to 4 weeks to determine seed yield.

### RT-qPCR analysis

WT plants at the prebolting stage were transferred to medium without or with 100 mM NaCl and cultured for 4 days. Total RNA was extracted from roots, rosette leaves, cauline leaves, the top half of the stem, buds (stages 6 to 12), flowers (stages 13 to 15), and siliques (stages 16 to 20) using TRI Reagent (Molecular Research Center Inc., OH, USA). cDNA synthesis was performed at 37°C for 15 min, 50°C for 5 min, and 98°C for 5 min using ReverTra Ace qPCR RT Master Mix with gDNA Remover (TOYOBO Co. Ltd., Osaka, Japan). RT-qPCR was performed with cDNA using StepOnePlus Real-Time PCR System (Applied Biosystems, USA). The thermal cycling condition were 30 s at 95°C, followed by 40 cycles of 5 s at 95°C and 30 s at 60°C. The polyubiquitin gene *UBQ10* (At4g05320) was used as a reference gene (*46*). The primers used were 5′-CCCTAACGGGAAAGACGATTAC-3′ and 5′-AGAGTTCTGCCATCCTCCAAC-3′ for *UBQ10* and 5′-CCTTAACATCACTCTCGAAGTTATC-3′ and 5′-AACCCATAACTCGCGTCTTT-3′ for *AtHKT1;1*.

### Histochemical localization

Plants at the prebolting stage were transferred to medium without or with 100 mM NaCl and cultured for 4 days. Plants were then fixed with 90% acetone for 15 min on ice and rinsed with 50 mM phosphate buffer (pH 7.0), and GUS staining was performed with 2 mM 5-bromo-4-chloro-3-indolyl-β-d-glucuronide (X-Gluc), 0.5 mM K_4_Fe(CN)_6_, 0.5 mM K_3_Fe(CN)_6_, and 50 mM phosphate buffer (pH 7.0) for 24 hours according to standard procedures (*47*). The reaction was stopped, and plants were destained in 70% ethanol. Samples were dehydrated with increasing concentrations of ethanol up to 100% and then embedded in Technovit 7100 (Heraeus Kulzer, Tokyo, Japan). The samples were sliced into 6-μm sections using a Leica RM2145 microtome (Leica, Wetzlar, Germany). Sections were stained with 0.01% neutral red (FUJIFILM Wako Chemicals, Japan) for 1 min, rinsed twice with water, sealed with Entellan new (FUJIFILM Wako Chemicals, Japan), and photographed.

### Immunoelectron microscopy

Two-week-old WT plants at the postbolting stage were transferred to hydroponic medium with 100 mM NaCl for 4 days. Stamens were collected from stage 15 flowers of each plant according to the protocol by Smyth *et al.* (*24*) and used for immunoelectron microscopy. The immunostaining was performed as described previously with some modifications (*19*, *48*). Briefly, the stamens were immersed in 50 mM phosphate buffer (pH 7.2) containing 0.5% glutaraldehyde and 4% paraformaldehyde at 4°C for 2 hours. After washing three times with phosphate buffer, the specimens were dehydrated using an ethanol series (50 to 99.5%, v/v), embedded in LR White resin (Electron Microscopy Sciences, PA, USA) and polymerized at −20°C under ultraviolet light for 2 days. Specimens were sectioned using a ULTRACUT-S ultramicrotome (Reichert-Nissei, Tokyo, Japan). Ultrathin sections (80 nm) were mounted on nickel grids and blocked with 4% BLOCK-ACE (KAC Co. Ltd., Kyoto, Japan) for 30 min at room temperature. Immunostaining with rabbit anti-AtHKT1;1 antiserum [rabbit immunoglobulin G (IgG); diluted 1:100] was performed on sections overnight at 4°C (*19*). After washing with blocking buffer (0.4% BLOCK-ACE), Alexa Fluor 546–FluoroNanogold Fab′ goat anti-rabbit IgG (#7404, Nanoprobes Inc., Yaphank, NY, USA) diluted in blocking buffer to 1:100 was added to the sections and incubated for 1 hour at room temperature. After washing with blocking buffer and distilled water, the sections were fixed with 1% glutaraldehyde in 50 mM tris-HCl (pH 7.5) containing 137 mM NaCl and 2.7 mM KCl, and the colloidal gold particles were treated with silver enhancement reagents (Nanoprobes Inc., NY, USA) (*49*). Last, the sections were stained with 4% uranyl acetate for 12 min in the dark, followed by incubation in Lead Stain Solution (18-0875; Sigma Chemical Co., St. Louis, USA) for 5 min, and then washed with distilled water. The stained sections were examined using a JEM-1400 transmission electron microscope (JEOL Ltd., Tokyo, Japan) at 100 kV.

### Micro-PIXE analysis

Two-week-old plants at the postbolting stage were transferred to medium with or without 75 mM NaCl and cultured for 3 days. Stamens were collected from stage 15 flowers. Micro-PIXE analysis was performed using a microbeam system, MB-I at Dynamitoron Laboratory in Tohoku University (*30*). Three–mega–electron volt proton beams were focused to 1 μm × 1 μm with a beam current of ca. 100 pA by a microbeam system that was composed of a microslit, a divergence slit, and a quadrupole doublet magnet (*30*). X-ray spectra and elemental maps via beam scanning over the sample were obtained using two Si (Li) x-ray detectors LS10129 and LS60148. To suppress background signals originating from the backing material the sample was held on a Cryofilm type IIC(9) (Section Lab, Hiroshima, Japan). At the downstream end of the chamber, the proton beam current was measured by a Faraday cup. Every PIXE dataset was obtained by 100,000 pC of dose for about 1 hour of measuring time, and then elemental maps were constructed using GeoPIXE II software.

### Production of ^43^K

Potassium-43 (^43^K) was produced by ^nat^Ca(γ,pxn) reactions using an electron linear accelerator at ELPH in Tohoku University. The calcium targets with oxide form were sealed in quartz tubes and irradiated with bremsstrahlung for several hours under water cooling. ^43^K produced was separated from the calcium target materials by oxalate precipitation and then isolated by cation exchange chromatography.

### Autoradiography measurements

Two-week-old plants at the postbolting stage were used for detection of ^22^Na and ^43^K absorption. For ^43^K absorption experiments, plants were incubated in 10 ml of hydroponic medium supplemented with 2 MBq of ^43^K for 15 hours in the light. Then, the plants were washed twice with hydroponic medium without ^43^K and separated into roots and shoots. For ^22^Na absorption experiments, plants were incubated in 10 ml of hydroponic medium containing 75 mM NaCl and 500 kBq of ^22^Na (Chiyoda Technol, Tokyo, Japan) for 2 days in the light. To keep the volume of the hydroponic medium constant at 10 ml, fresh hydroponic medium containing 75 mM NaCl was added every 12 hours. Then, the plants were washed with the hydroponic medium containing 75 mM NaCl twice and separated into roots and shoots. The labeled plant materials were then put onto imaging plates (FUJIFILM, Tokyo, Japan) and exposed at 4°C for 8 hours. The imaging plates were scanned in a Typhoon FLA 9500 laser scanner (GE Healthcare Japan K.K., Tokyo, Japan).

### Quantitative analysis of ^22^Na or ^43^K by γ-ray spectrometry

Following the autoradiography measurements, the labeled shoots and roots were further separated into stem, cauline leaf, rosette leaf, shoot base, root, bud (stages 6 to 12), flower (stages 13 to 15), and silique (stages 16 to 20) and were collected into separate tubes to determine their fresh weight. The amounts of ^22^Na or ^43^K radionuclides in these different plant parts were then measured by γ-ray spectrometry with a high-purity germanium semiconductor detector of 35% relative efficiency. The samples were placed at 2 mm from the surface of the detector in a lead-shield box. The peak areas at 1275 keV (for ^22^Na) and 373 keV (for ^43^K) were used to quantify the amount of ^22^Na and ^43^K radionuclides present in the samples.

### Two-electrode voltage clamp recordings using *Xenopus laevis* oocytes

Oocytes were surgically isolated from mature female *X. laevis*, enzymatically defolliculated, and stored at 18°C in Barth’s buffer. Capped mRNA coding for *AtHKT1;1*, *AtHKT1;1^S68G^*, *TaHKT2;1*, and *TsHKT1;2* was synthesized using the mMESSAGE mMACHINE T7 Transcription Kit (Thermo Fisher Scientific). Approximately 10 ng of mRNA was injected per oocyte and activities were recorded 2 days after injection. After the oocytes were impaled with glass micropipette electrodes filled with 3 M KCl, voltage clamp protocol was applied as previously described (*50*). Voltage-clamp application and data analysis were carried out using an AxoClamp 2B amplifier (Axon Instruments), Axon Digidata 1550 System (Molecular Devices) and Clampex software (*51*). The bath buffer contained 1 mM MgCl_2_, 1 mM CaCl_2_, 10 mM Hepes-NaOH (pH 7.3), and either KCl or NaCl was added at the concentrations indicated in the figures (*34*).

### Plant hormone quantification

Plants at the prebolting stage were transferred to hydroponic medium with or without 30 mM NaCl and grown for an additional two weeks. Stage 12–14 and 15–16 flowers were collected from each plant. Extraction, purification and quantification of plant hormones by LC-MS/MS were performed as described previously (*52*).

### Statistical analysis

Prism 6.01 software (GraphPad) was used for statistical analyses: two-tailed Student’s *t* test to determine differences between two groups and one-way analysis of variance (ANOVA) with Tukey-Kramer test for multiple-group comparisons.
