# Supplementary material for: The HKT1 Na+ transporter protects plant fertility by decreasing Na+ content in stamen filaments
Source: Sci Adv. 2023 Jun 2;9(22):eadg5495. doi: 10.1126/sciadv.adg5495 (PMC10413666; doi:10.1126/sciadv.adg5495)
Supplement: Supplementary file 1 — Figs. S1 to S11 [file sciadv.adg5495_sm.pdf]

Science Advances
AAAS

Supplementary Materials for

The HKT1 Na^+ transporter protects plant fertility by decreasing Na^+ content in stamen filaments

Takeshi Uchiyama *et al.*

Corresponding author: Nobuyuki Uozumi, uozumi@tohoku.ac.jp

Sci. Adv. **9**, eadg5495 (2023)
DOI: 10.1126/sciadv.adg5495

This PDF file includes:

Figs. S1 to S11

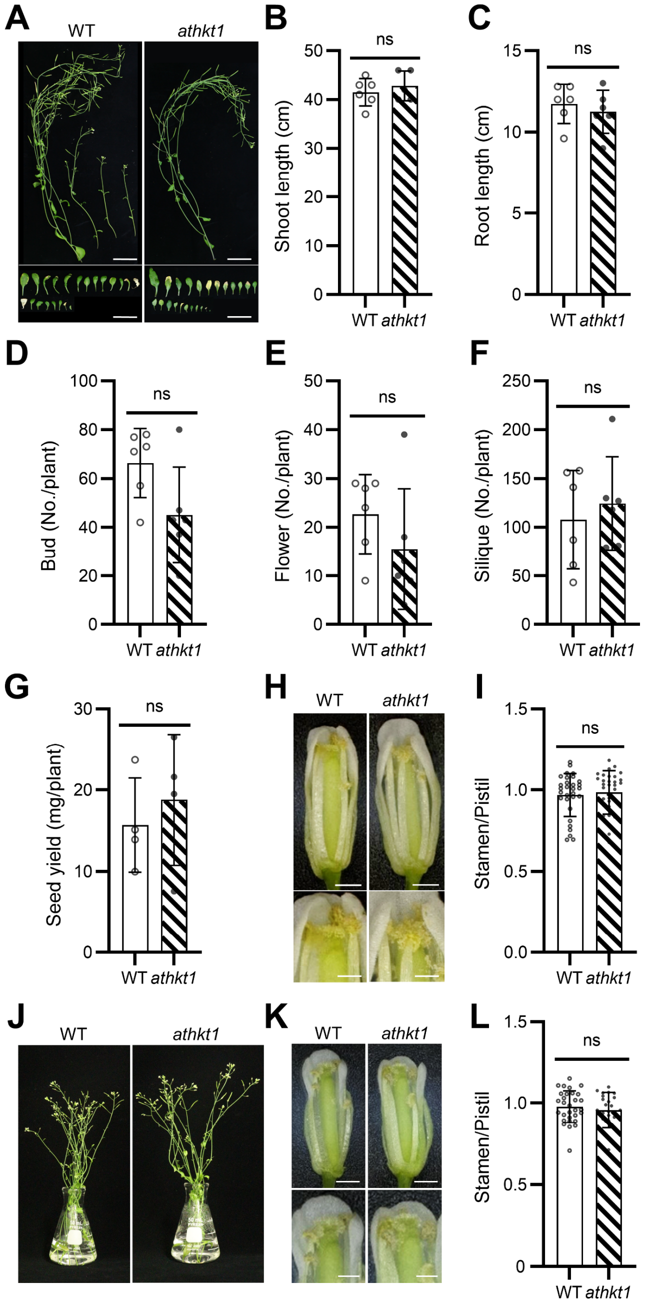

Fig. S1. Phenotypes of flowers from the *athkt1* mutant under control conditions (without added Na^+).

(**A**) Shoot and rosette leaves of a typical plants, the wild type (WT) and the *athkt1* mutant, are shown. Scale bar = 3 cm. (**B**) Shoot length, (**C**) root length, number of (**D**) buds, (**E**) flowers and (**F**) siliques. Data are from three independent experiments. Values are the mean ± S.D.; $n = 6$; Student's *t*-test, $*P < 0.05$; ns, not significant. (**G**) Seed yield. Data are from three independent experiments. $n = 4$. (**H**) Phenotype of flowers of the wild type and the *athkt1* mutant. Scale bar = 500 μm (top panel) and 250 μm (bottom panel). (**I**) Ratio of length of stamens to pistil of the plants shown in panel (H). $n = 30$. (**J**) Phenotypes of cut flower stalks. (**K**) Phenotypes of flowers from

excised flower stalks shown in panel (J). Scale bar = 500 µm (top panel) and 250 µm (bottom panel). (**L**) Ration of length of stamens to pistil of flower stalks shown in panel (J). *n* = 18-30.

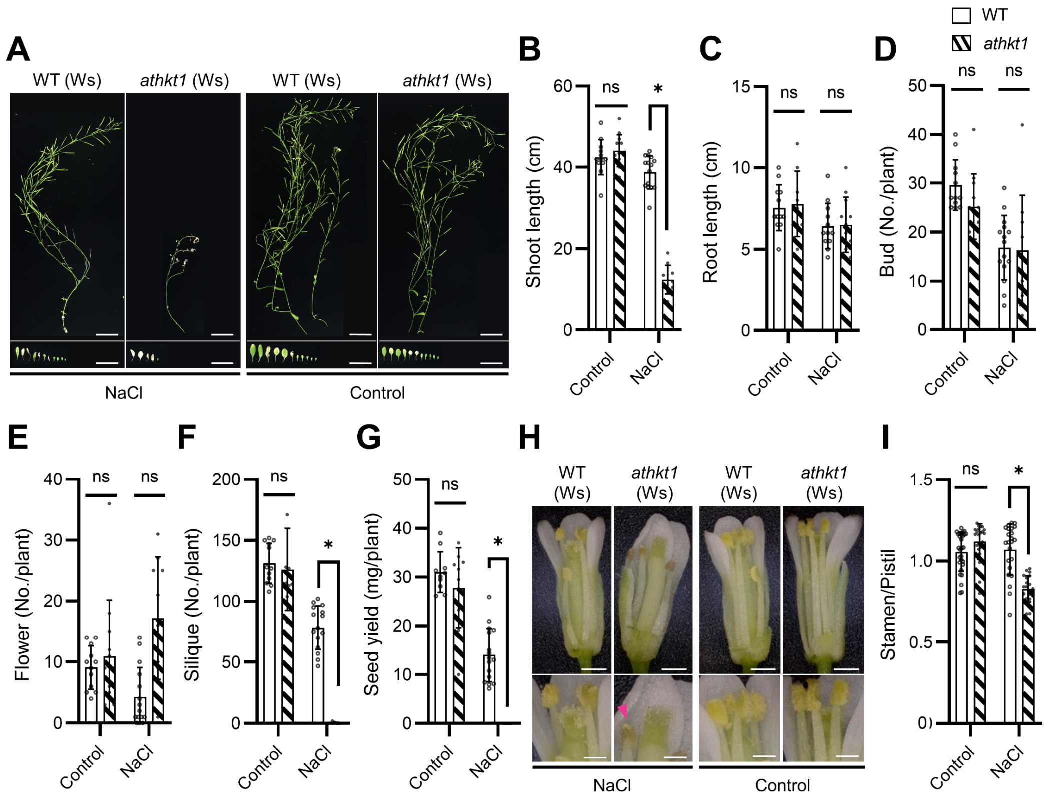

Fig. S2. Phenotypes of the *athkt1* mutant in the Ws background.

All experiments (A to I) were performed the same as those in Fig. 1 (A to I). (**A**) Salt stress treatment of the wild type (WT) and the *athkt1* mutant in the Ws background. NaCl (30 mM) was added one day before bolting and the plants were grown for three more weeks. Shoot and rosette leaves of a typical plant are shown. Scale bar = 3 cm. (**B**) Shoot length, (**C**) root length, number of (**D**) buds, (**E**) flowers, and (**F**) siliques. Data are presented as mean ± S.D.; n = 10-15; Student's t-test, *$P < 0.05$; ns, not significant ($P > 0.05$). (**G**) Seed yield. Data are presented as mean ± S.D.; n = 10-16; Student's t-test, *$P < 0.05$; ns, not significant ($P > 0.05$). (**H**) Phenotype of flowers of the wild type and the *athkt1* mutant. NaCl (30 mM) was added one day before bolting and the plants were grown for two more weeks. The magenta arrowhead points out the short stamen. Scale bar = 500 μm (top panel) and 250 μm (bottom panel). (**I**) Ratio of length of stamens to pistil of plants shown in panel (h). Data are presented as mean ± S.D.; n = 20-28; Student's t-test, *P < 0.05; ns, not significant ($P > 0.05$).

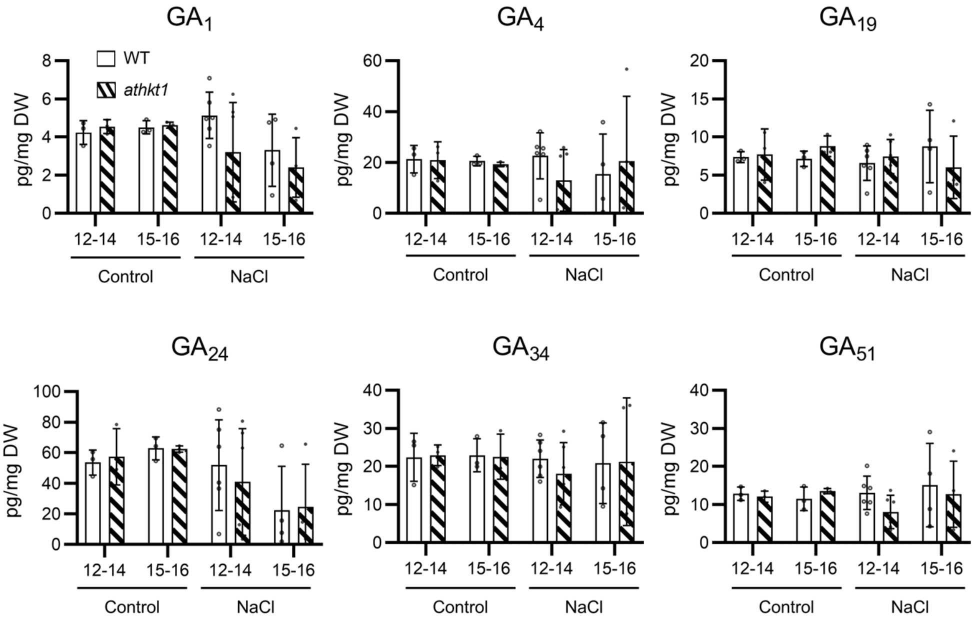

Fig. S3. Quantification of gibberellin content in *athkt1* flowers.

GA_1, GA_4, GA_{19}, GA_{24}, GA_{34} and GA_{51} content was determined by LC-MS/MS in wild-type (WT) and mutant flowers harvested at either stage 12-14 or stage 15-16. 30 mM NaCl was added one day before bolting and plants were grown for two more weeks. Data are presented as mean ± S.D.; n = 3-6.

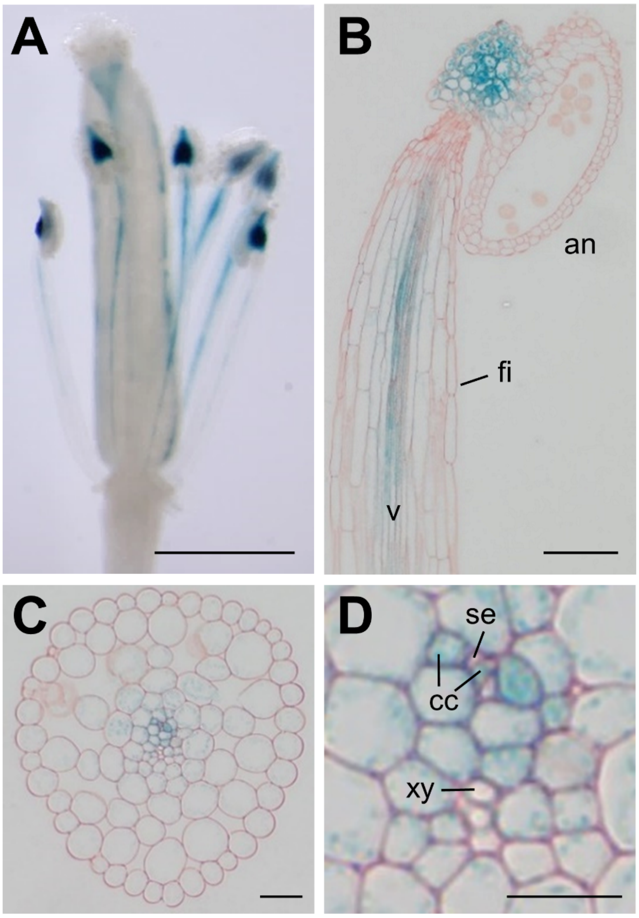

Fig. S4. Localization of AtHKT1;1 in floral organs under control conditions (without added Na^+).
(**A** to **D**) GUS staining of the flowers without sepals and petals (A), stamen (B), cross-section of filament (C). (D) Magnification of part of the filaments in panel (C). an, anther; fi, filament; se, sieve element; cc, companion cell; ph, phloem; xy, xylem; v, vascular bundle. Scale bars are 1 mm for (A); 100 μm for (B); 20 μm for (C); 10 μm for (D).

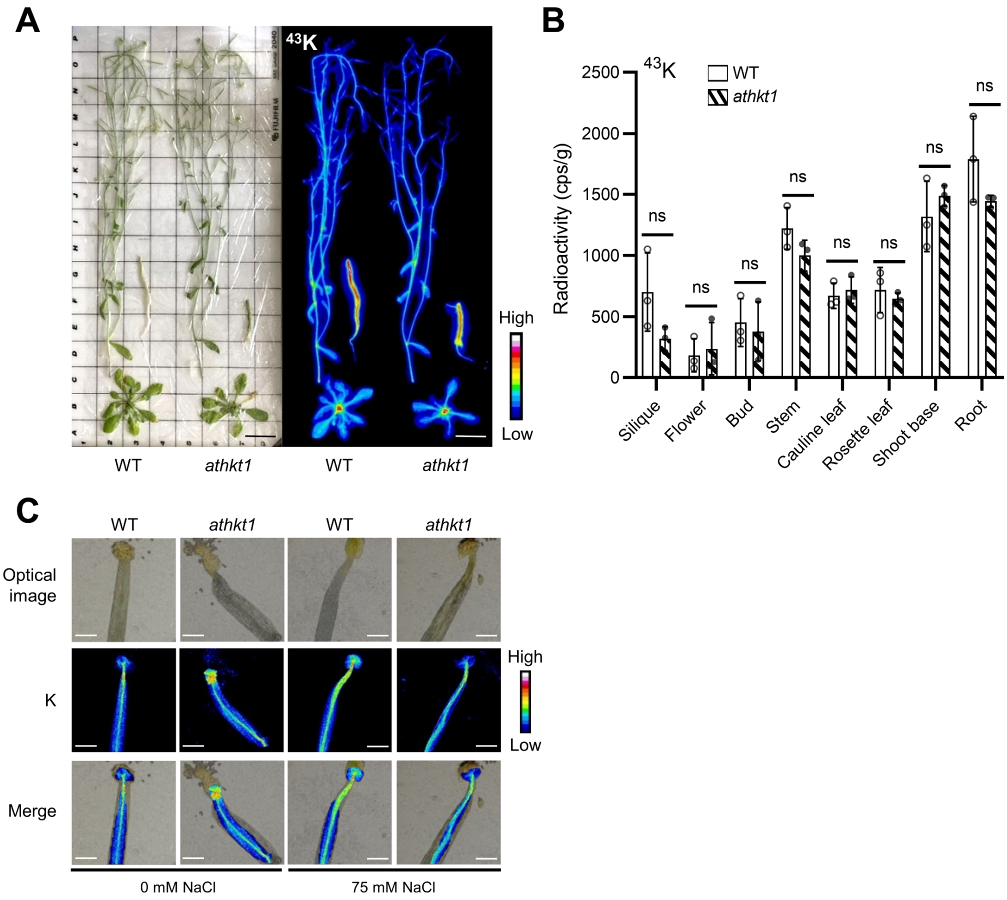

Fig. S5. Distribution of K^+ during the reproductive stage of the *athkt1* mutant.
(**A**) Representative autoradiographs of the wild type and the *athkt1* mutant incubated with ^{43}K. Scale bars = 3 cm. (**B**) Quantification of the radioisotopes in various organs in ^{43}K treated plants. Data are from three independent experiments. $n = 3$ for the panel (A). (**C**) Elemental maps of K in stamens of the wild type and the *athkt1* mutant cultured as in panel (A) obtained by micro-PIXE analysis. Scale bars = 200 μm. Data are from three independent experiments.

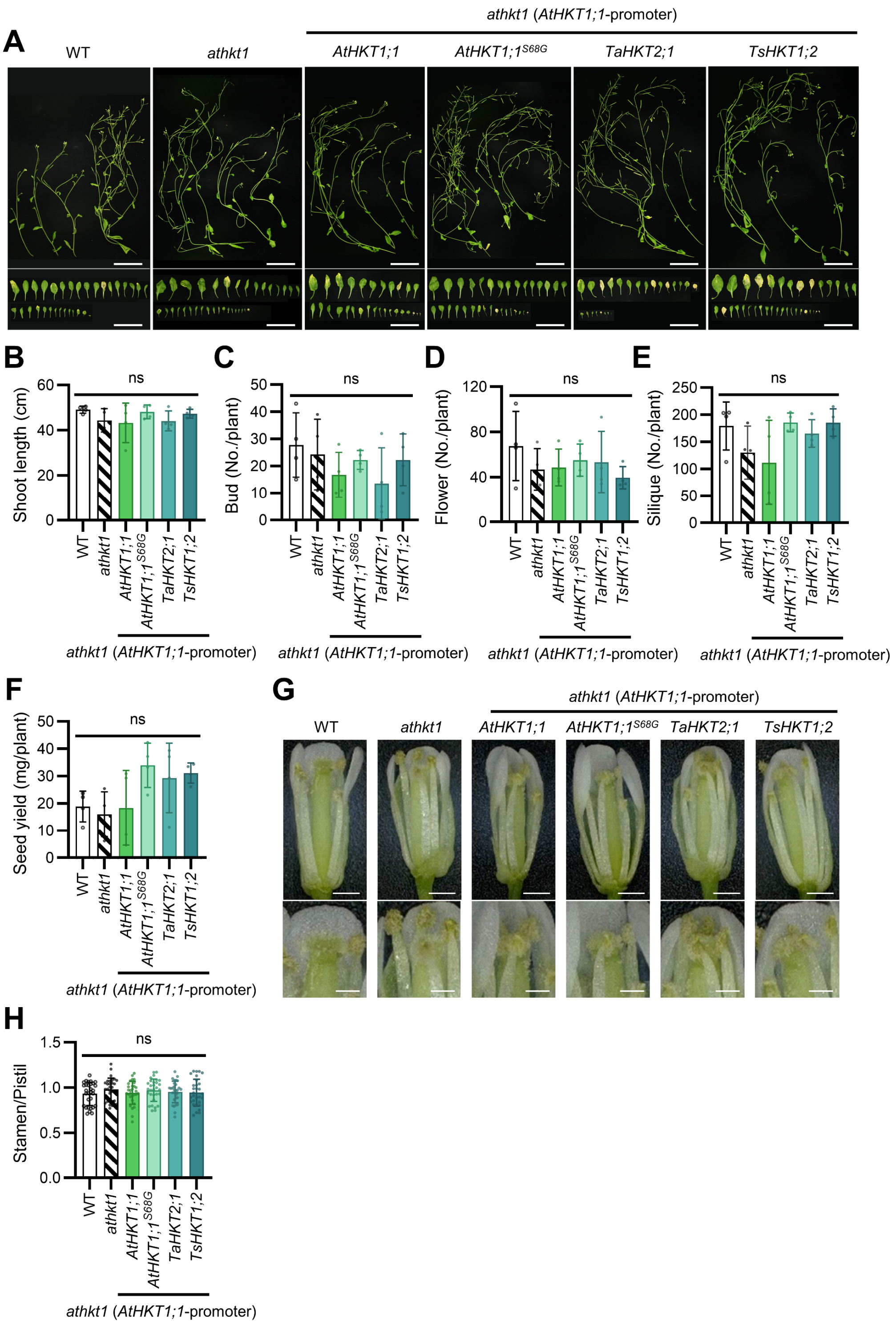

Fig. S6. Phenotype of the *athkt1* mutant transformed with different HKTs under control conditions (without added Na$^+$).

(**A**) Phenotype of the wild type (WT), *athkt1* mutant and $P_{AtHKT1;1}$-*HKT*s/*athkt1*. Shown are shoot and rosette leaves of a typical plant. Scale bar = 5 cm. (**B** to **E**) Shoot length (B), number of buds

(C), flowers (D) and siliques (E) are shown. Data are from three independent experiments. Values are the mean ± S.D.; n = 4-5; one-way ANOVA with Tukey-Kramer test, $P < 0.05$; ns, not significant. (**F**) Seed yield. Data are from three independent experiments. n = 4-6. (**G**) Phenotypes of the flowers. Scale bar = 500 µm (top panel) and 250 µm (bottom panel). (**H**) Ratio of length of stamens to pistil of plants shown in panel (G). n = 30.

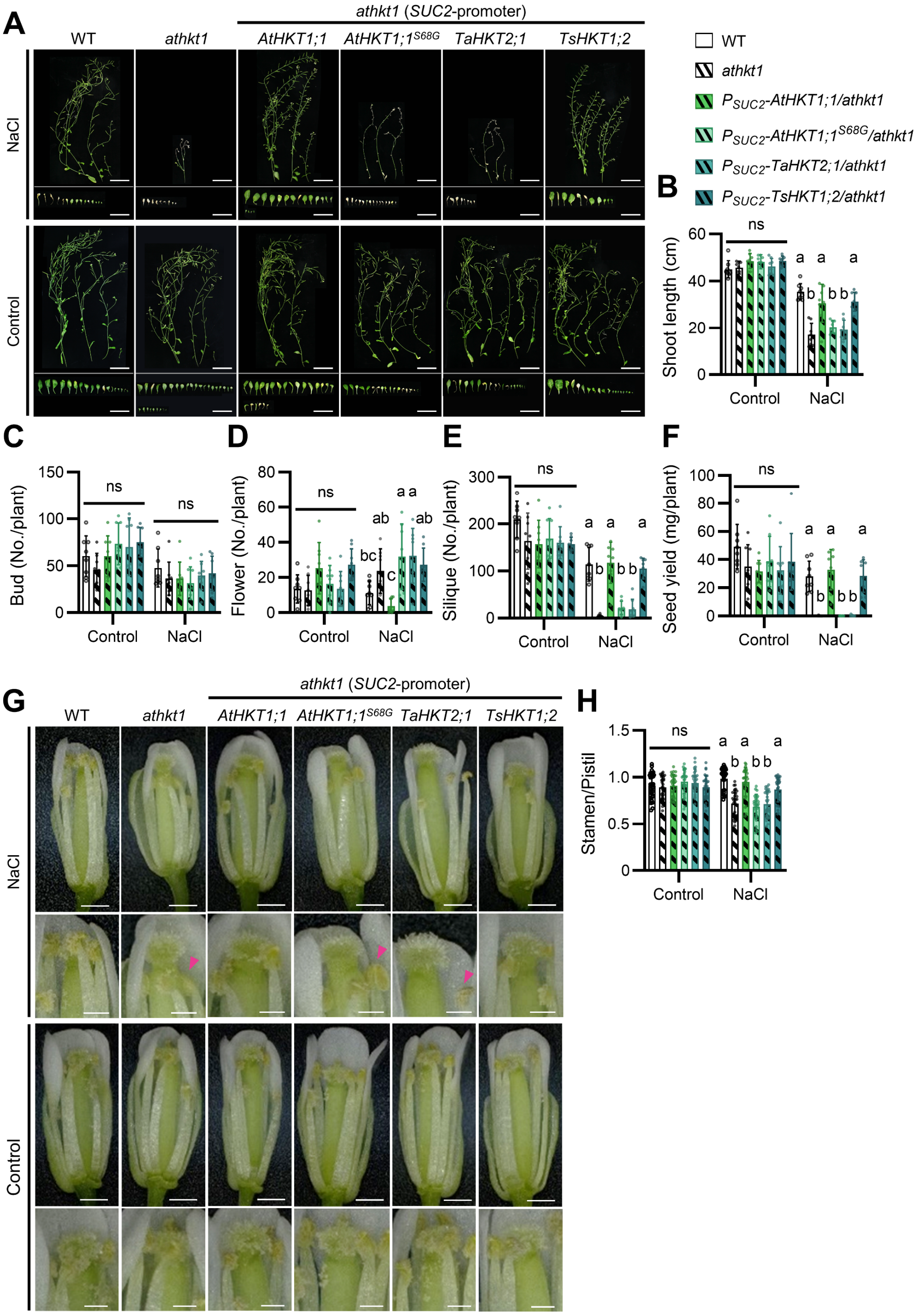

Fig. S7. Phenotype of the *athkt1* mutant expressing HKTs in companion cells.
(**A**) The wild type (WT), the *athkt1* mutant and P_{SUC2}-*HKT*s/*athkt1* (AtHKT1;1, AtHKT1;1^{S68G}, TaHKT2;1 and TsHKT1;2) were grown hydroponically, NaCl (30 mM) was added one day before bolting and plants were grown for three more weeks. Shown are shoot and rosette leaves of a typical plant. Scale bar = 5 cm. (**B** to **F**) Shoot length (B) and number of buds (C), flowers (D),

siliques (E) and seed yield (F) are shown. Data are from three independent experiments. Values are the mean ± S.D.; $n = 8$; one-way ANOVA with Tukey-Kramer test, $P < 0.05$; ns, not significant ($P > 0.05$). (**G**) Phenotype of the flowers. NaCl (30 mM) was added one day before the bolting and the plants were grown for two more weeks. The magenta arrowheads point out the short stamens. Scale bar = 500 µm (top panel) and 250 µm (bottom panel). (**H**) Ratio of length of stamens to pistil of plants shown in panel (G). Data are from three independent experiments. Values are the mean ± S.D.; $n = 30$; one-way ANOVA with Tukey-Kramer test, $P < 0.05$; ns, not significant ($P > 0.05$).

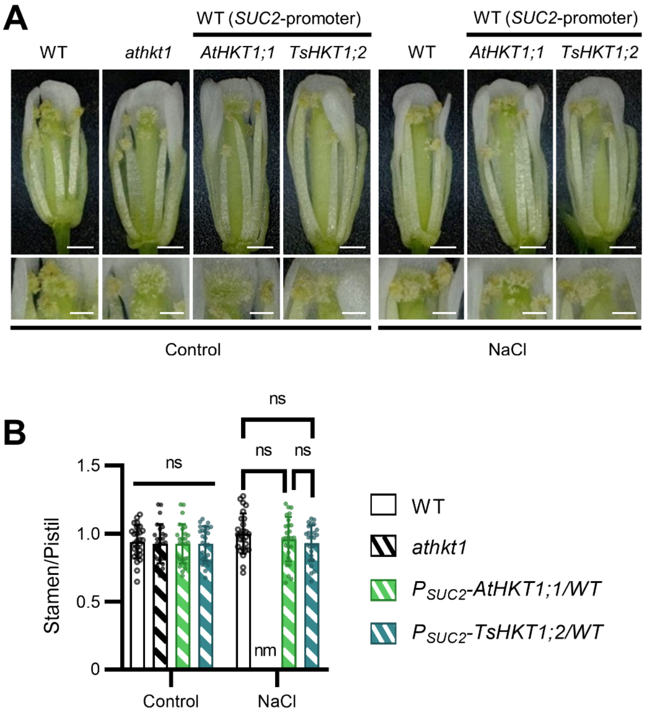

Fig. S8. Phenotype of flower of the wild type expressing HKTs in companion cells.
(**A**) Phenotype of flowers of the wild type, the *athkt1* mutant and P_{SUC2}-*HKT*s/WT (AtHKT1;1 or TsHKT1;2). Plants were grown hydroponically, then, starting the day before bolting, the NaCl concentration was increased stepwise (30, 50, 75 mM) every five days for a total of 15 days. Scale bar = 500 µm (top panel) and 250 µm (bottom panel). (**B**) Relative length of stamens to pistil of plants shown in panel (A). Data are from three independent experiments. Values are the mean ± S.D.; $n = 26$; one-way ANOVA with Tukey-Kramer test, $P < 0.05$; ns, not significant ($P > 0.05$); nm, not measured.

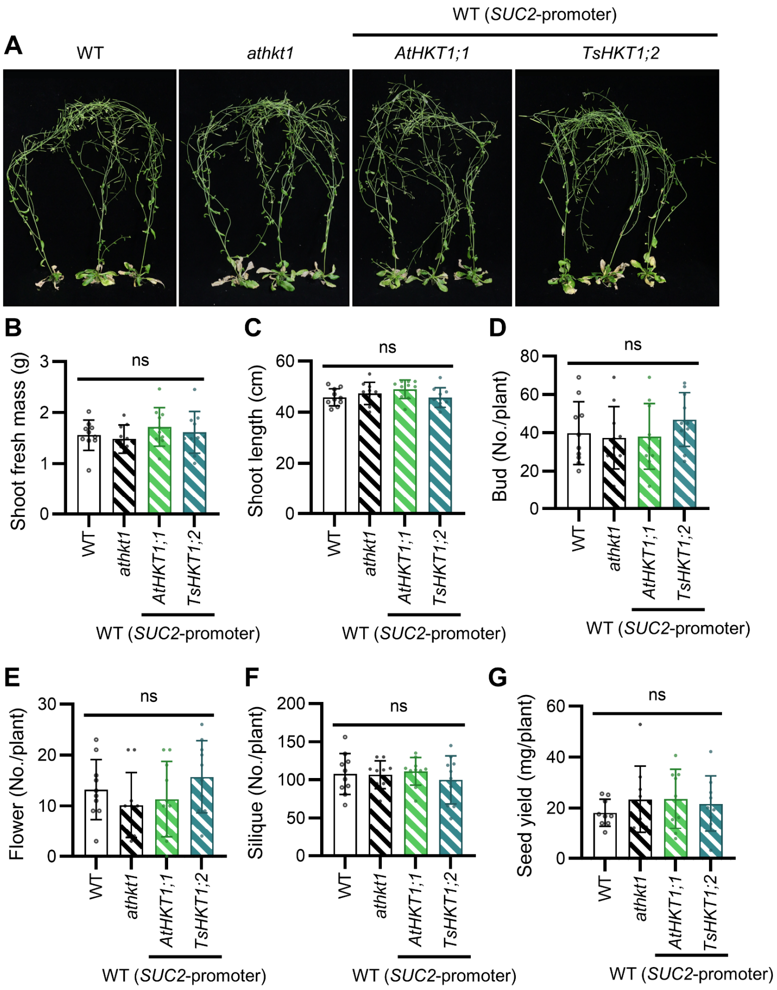

Fig. S9. Phenotype of the wild type expressing HKTs in companion cells under control conditions (without added Na⁺).

(**A**) Phenotype of the wild type (WT), the *athkt1* mutant and *P*$_{SUC2}$-*HKT*s/WT. Shown are typical plants. (**B**) Fresh weight of shoots of plants grown in saline conditions. Data are from three independent experiments. *n* = 10-13. (**C** to **F**) Shoot length (C) and number of buds (D), flowers (E) and siliques (F) are shown. *n* = 10-13. (**G**) Seed yield. *n* = 9-12.

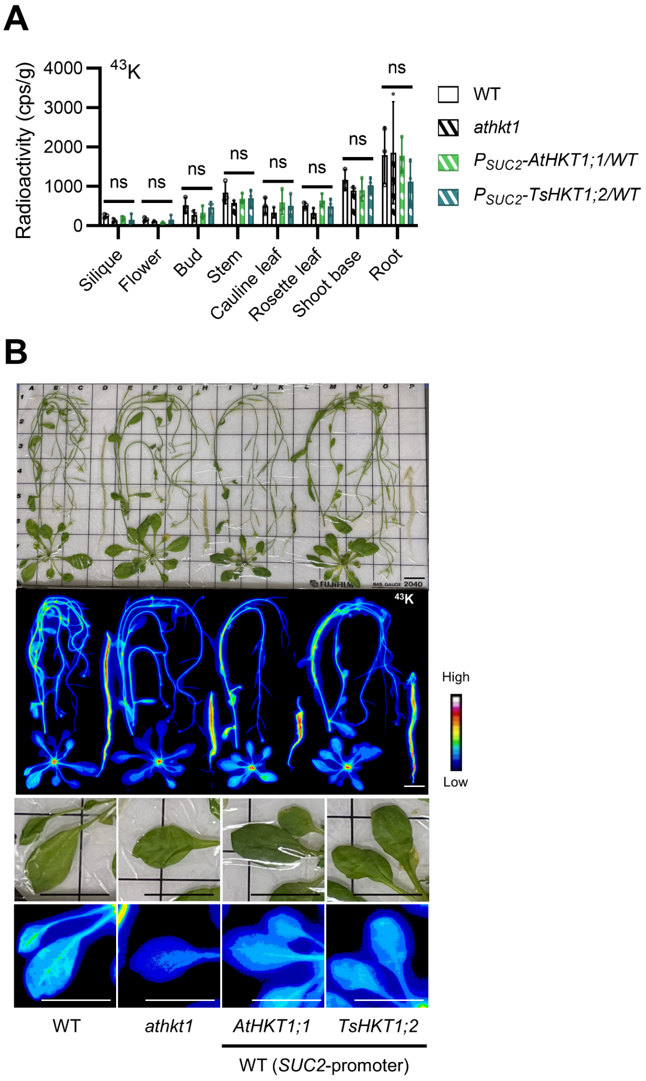

Fig. S10. Distribution of K⁺ during the reproductive stage of the wild type expressing HKTs in companion cells.
(**A**) Radioactivity of siliques, flowers, buds, stems, cauline leaves, rosette leaves, shoot bases and roots in ^{43}K treated plants shown in panels (B). Data are presented as mean ± S.D.; $n = 4$; one-way ANOVA with Tukey-Kramer test; $P < 0.05$, ns, not significant ($P > 0.05$). (**B**) Representative autoradiographs of the wild type (WT), the *athkt1* mutant and P_{SUC2}-*HKT*s/WT (AtHKT1;1 or TsHKT1;2) treated with ^{43}K. Scale bars = 3 cm.

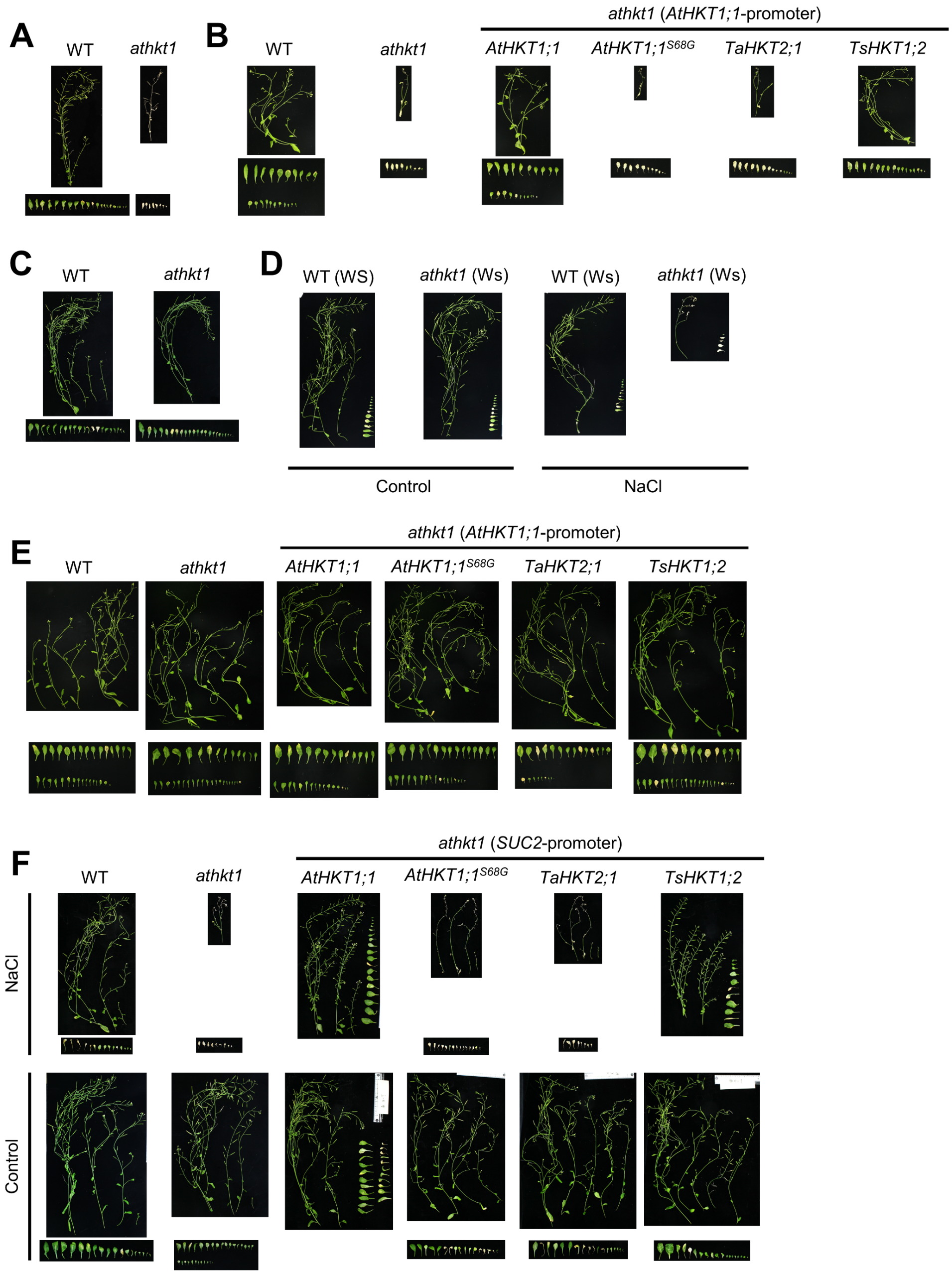

Fig. S11. Original images of plant phenotypes used to create (A) Fig. 1, (B) Fig. 4, (C) Fig. S1, (D) Fig. S2, (E) Fig. S6 and (F) Fig. S7.
